# Supplementary material for: The Effects of Rapid Mitochondrial Gene Loss on Organellar Proteomes
Source: Genome Biol Evol. 2026 Jun 17;18(6):evag147. doi: 10.1093/gbe/evag147 (PMC13308715; doi:10.1093/gbe/evag147)
Supplement: evag147_Supplementary_Data [file evag147_supplementary_data.pdf]

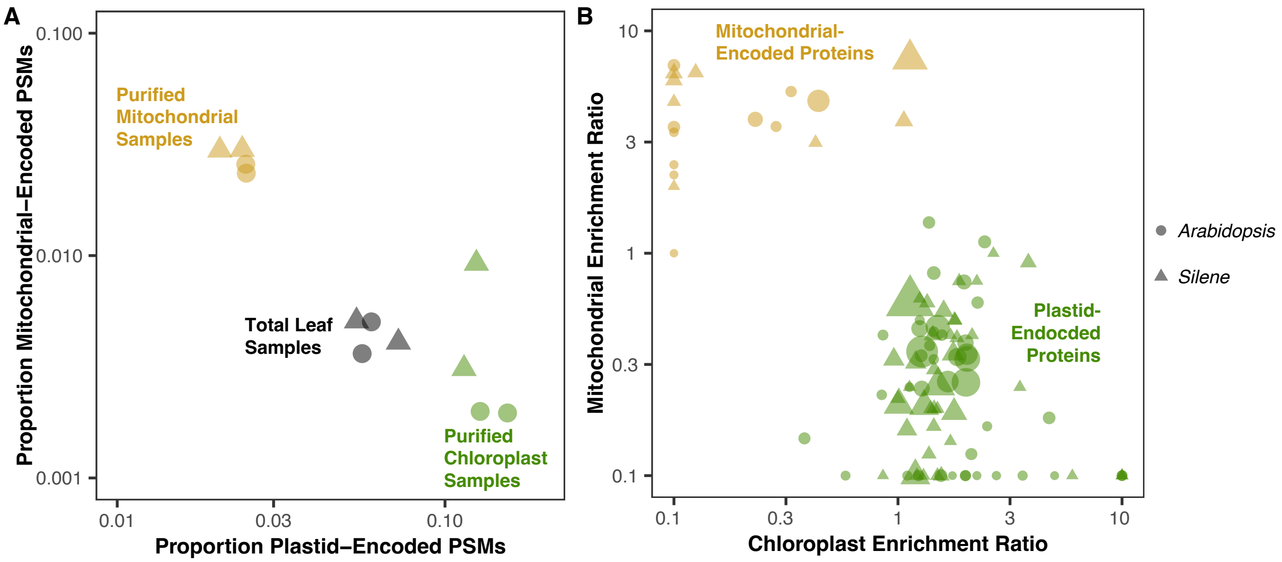

Figure S1. Enrichment of proteins encoded by the mitochondrial and plastid genomes in purified organelle fractions. (A) In this panel, each point represents an individual biological sample, showing its cumulative number of PSMs across all plastid-encoded proteins (x-axis) and mitochondrial-encoded proteins (y-axis) expressed as a proportion of all PSMs in the sample with each biological replicate shown separately. (B) In this panel, each point represents a mitochondrial-encoded or plastid-encoded protein. Enrichment in chloroplast samples (x-axis) or mitochondrial samples (y-axis) is calculated by dividing the PSM count for that protein from the respective purified organelles by the corresponding PSM count from total leaf samples. Point size is scaled based on total number of PSMs for that protein across all samples. Only proteins represented by at least 5 unique peptides in the dataset are shown, and enrichment/depletion values were capped at 10-fold for visualization purposes. The two biological replicates are averaged for this panel. In both panels, point shape indicates species identity (circle: *A. thaliana*; triangle: *S. conica*), and PSMs were excluded for peptides shared between multiple proteins.

***Arabidopsis thaliana* PSMs**

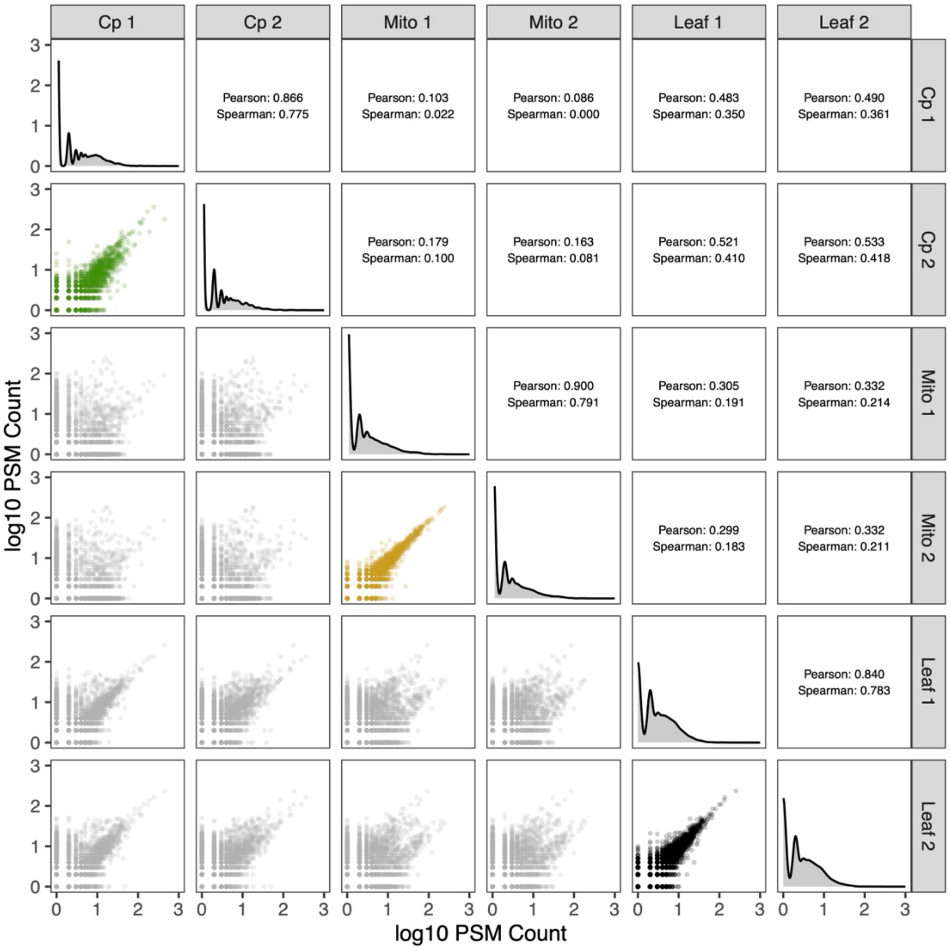

Figure S2. Pairwise correlation matrix among the six *A. thaliana* samples based on PSMs for each identified protein. Each point in the scatterplots below the downward diagonal represents a protein. The highlighted subplots show the three pairs of biological replicates of the same sample type that are expected to have high correlations. The correlation coefficients (Pearson's *r* and Spearman's ρ) corresponding to each subplot are shown above the downward diagonal. The downward diagonal itself shows the univariate distribution (density kernel) for PSM counts in each of the six samples. PSMs were excluded for peptides that were shared between multiple proteins. A baseline value of 1 was added to all values prior to log-transformation and visualization.

***Arabidopsis thaliana* Ion Intensities**

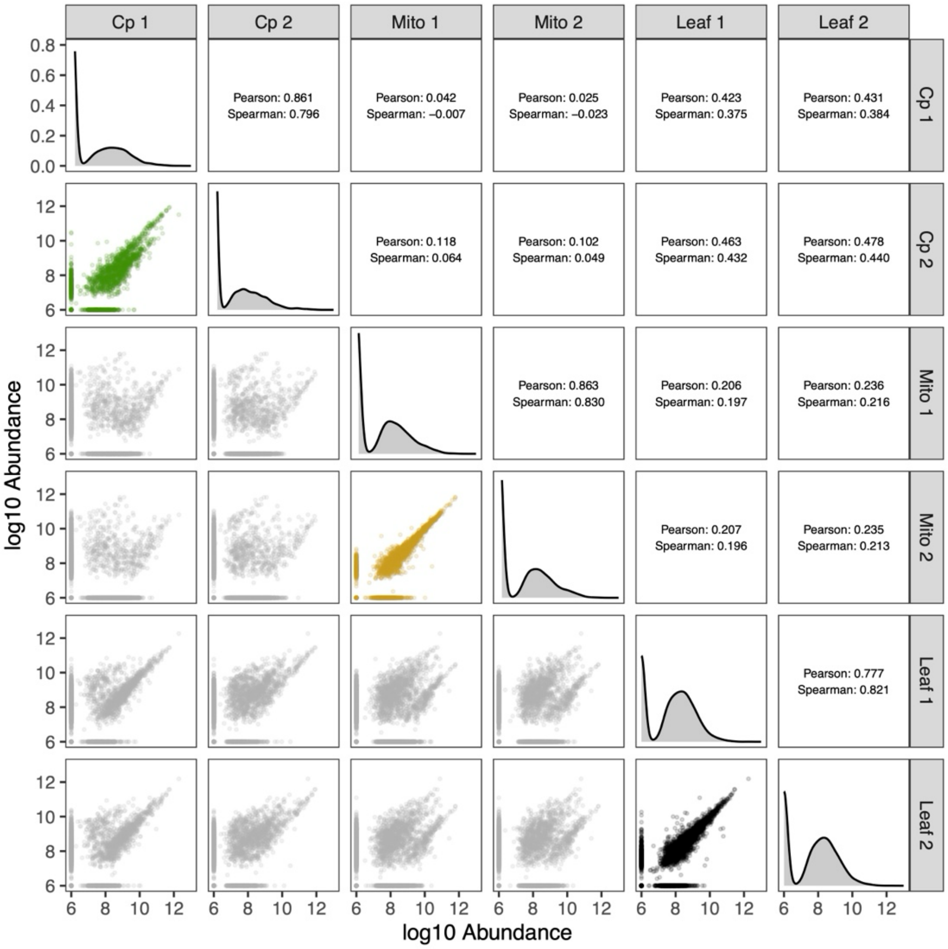

Figure S3. Pairwise correlation matrix among the six *A. thaliana* samples based on ion intensity for each identified protein. Each point in the scatterplots below the downward diagonal represents a protein. The highlighted subplots show the three pairs of biological replicates of the same sample type that are expected to have high correlations. The correlation coefficients (Pearson's *r* and Spearman's ρ) corresponding to each subplot are shown above the downward diagonal. The downward diagonal itself shows the univariate distribution (density kernel) for ion intensities in each of the six samples. Ion intensities were excluded for peptides that were shared between multiple proteins or that were identified solely based on an MS1 peak that was not validated in that sample with an MS2 spectrum. A baseline value of 1e6 was added to all values prior to log-transformation and visualization.

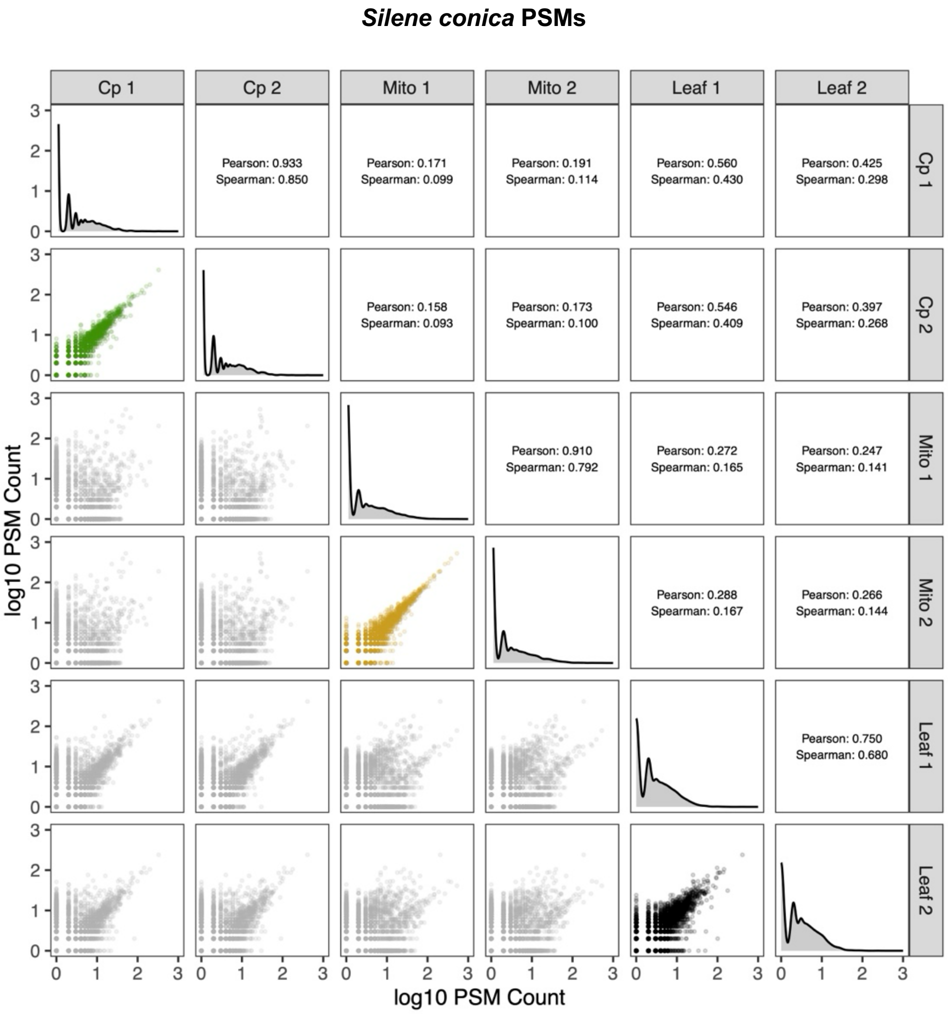

Figure S4. Pairwise correlation matrix among the six *S. conica* samples based on PSMs for each identified protein. Each point in the scatterplots below the downward diagonal represents a protein. The highlighted subplots show the three pairs of biological replicates of the same sample type that are expected to have high correlations. The correlation coefficients (Pearson's r and Spearman's ρ) corresponding to each subplot are shown above the downward diagonal. The downward diagonal itself shows the univariate distribution (density kernel) for PSM counts in each of the six samples. PSMs were excluded for peptides that were shared between multiple proteins. A baseline value of 1 was added to all values prior to log-transformation and visualization.

***Silene conica* Ion Intensities**

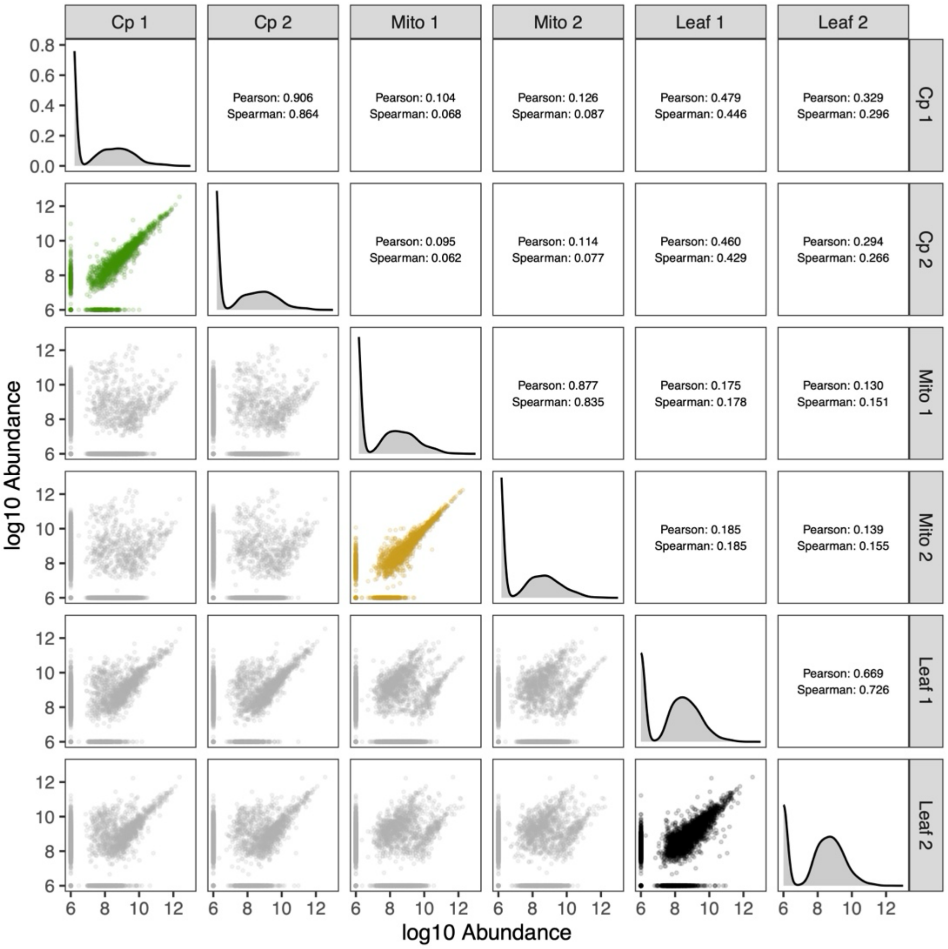

Figure S5. Pairwise correlation matrix among the six *S. conica* samples based on ion intensity for each identified protein. Each point in the scatterplots below the downward diagonal represents a protein. The highlighted subplots show the three pairs of biological replicates of the same sample type that are expected to have high correlations. The correlation coefficients (Pearson's *r* and Spearman's ρ) corresponding to each subplot are shown above the downward diagonal. The downward diagonal itself shows the univariate distribution (density kernel) for ion intensities in each of the six samples. Ion intensities were excluded for peptides that were shared between multiple proteins or that were identified solely based on an MS1 peak that was not validated in that sample with an MS2 spectrum. A baseline value of 1e6 was added to all values prior to log-transformation and visualization.

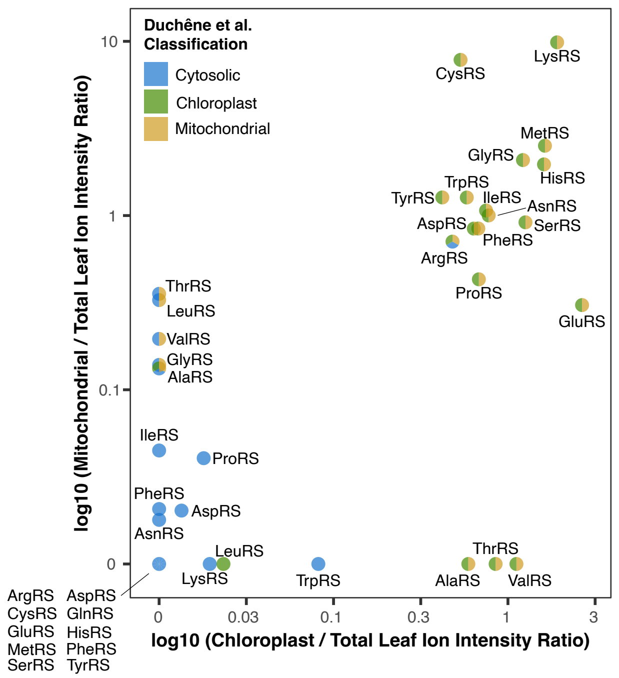

Figure S6. Summary of mitochondrial and chloroplast enrichment of *Arabidopsis thaliana* aaRSs relative to total leaf samples based on ratios of ion intensity averaged across two biological replicates. Ion intensities were excluded for peptides that were shared between multiple proteins or if they were identified solely based on an MS1 peak that was not validated in that sample with an MS2 spectrum. Color coding of points reflects whether the aaRS was previously classified as being targeted to the cytosol, chloroplasts, and/or mitochondria (Duchêne et al. 2005; Duchêne et al. 2009).

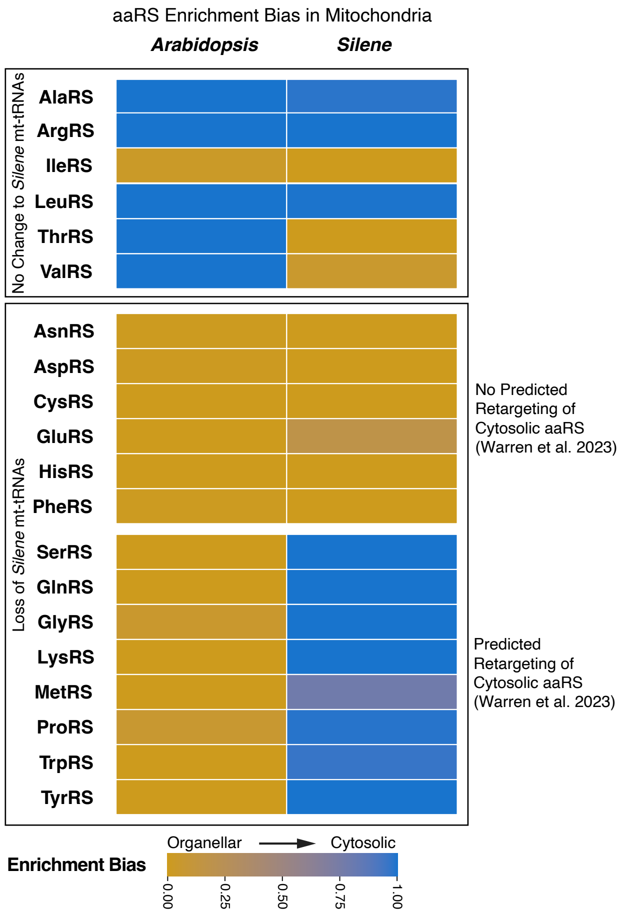

Figure S7. Summary of whether mitochondrial samples were biased towards containing organellar-like vs. cytosolic-like aaRSs based on proteomic analysis. This is the same representation as Figure 4A except that enrichment bias was calculated using ion intensity rather than PSM counts as the quantification metric. ion intensities were excluded for peptides that were shared between multiple proteins or if they were identified solely based on an MS1 peak that was not validated in that sample with an MS2 spectrum. See Methods and Figure 4 legend for more information.

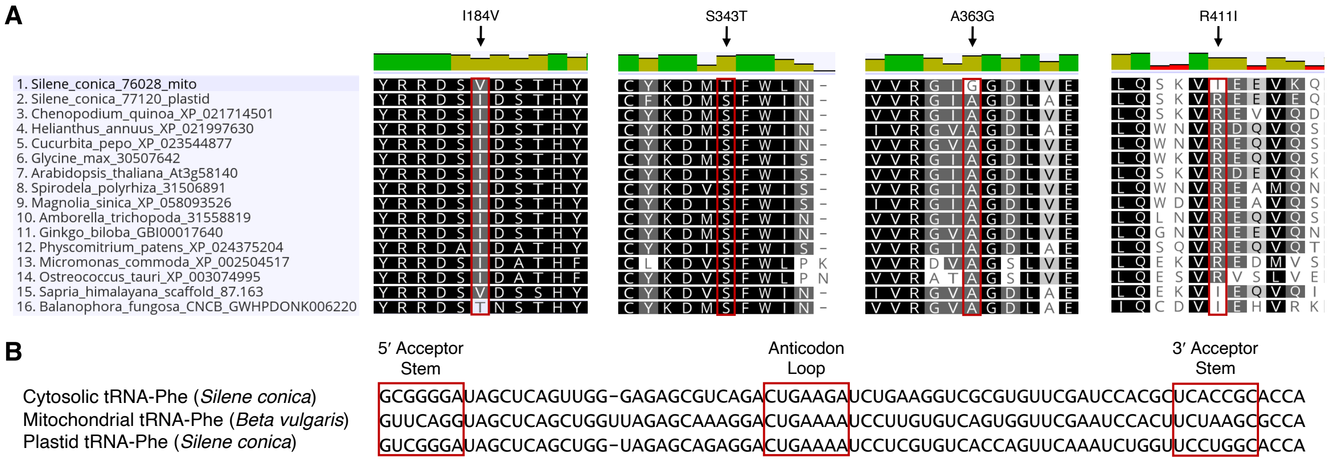

Figure S8. PheRS and tRNA-Phe alignments. (A) Amino acid alignments showing four positions in the PheRS sequence that have a derived change in the *Silene conica* mitochondrial PheRS but are otherwise conserved across a diverse sampling of green plants/algae. In two of these cases (I184V and R411I), parallel substitutions are observed in the parasitic plant taxa *Sapria himalayana* and *Balanophora fungosa*. (B) Alignment of cytosolic, mitochondrial, and plastid tRNA-Phe.

Table S1. Abundance of SUBA5-classified mitochondrial and plastid proteins in *A. thaliana* LC-MS/MS samples. The reported percentages reflect the total ion intensity or PSMs for proteins in the respective SUBA5 category divided by the total ion intensity or PSM signal for all identified proteins.

	SUBA5 Mitochondrial Proteins			SUBA5 Plastid Proteins		
**Arabidopsis Sample**	**# Detected**	**Ion Intensity**	**PSMs**	**# Detected**	**Ion Intensity**	**PSMs**
Mitochondrial 1	835	73.96%	59.93%	459	9.79%	14.21%
Mitochondrial 2	783	73.16%	61.25%	363	10.15%	13.27%
Leaf 1	459	6.79%	11.29%	1110	70.07%	41.58%
Leaf 2	449	8.18%	12.48%	1090	69.65%	43.07%
Chloroplast 1	176	2.06%	5.25%	1314	96.21%	91.55%
Chloroplast 2	161	1.78%	4.74%	1138	95.54%	86.56%

Table S2. Normalized MS1 ion intensities for PheRS enzymes in *S. conica*.

Sample	PheRS Type Cytosolic (Sconica_v3_11405-RA)	PheRS Type Chloroplast (Sconica_v3_09891-RA)	PheRS Type Mitochondrial (Chr01-anno1.g64625.t1)	Ratio of Mito to Chloro Types[b]
Mt1	1.03e8	6.25e8	1.83e9	2.93
Mt2	9.70e7	5.21e8	1.39e9	2.67
Cp1	2.77e8	2.35e9	1.28e8	0.05
Cp2	5.51e8	3.35e9	3.19e8	0.10
Lf1	1.45e9	2.81e9	1.87e8	0.07
Lf2	1.58e9	2.37e9	5.30e8	0.22

[a] Reported values exclude contributions from shared peptides but do include intensities reported only as "Peak Found" without PSM support. The identified differences would be even larger if these were excluded, as the putative mitochondrial PheRS produced no PSMs outside of the mitochondrial samples.

[b] The mitochondrial samples have a significantly higher ratio of mitochondrial to chloroplast PheRS types than the chloroplast samples (one-sided Student's *t-test* of log-normalized ratios; $p = 0.0029$).

Table S3. PSM counts by sample for additional enzymes involved in tRNA metabolism in mitochondria and other compartments

	Arabidopsis thaliana							***Silene conica***						
**Enzyme**	**AGI ID**	**Mt1**	**Mt2**	**Cp1**	**Cp2**	**Leaf1**	**Leaf2**	**Gene ID**	**Mt1**	**Mt2**	**Cp1**	**Cp2**	**Leaf1**	**Leaf2**
GatA	AT3G25660	6	7	7	2	6	3	Sconica_v3_10523-RA	0	0	6	10	3	2
GatB	AT1G48520	8	7	12	3	7	5	Sconica_v3_48968-RA	0	0	12	15	5	1
GatC	AT4G32915	4	3	2	1	0	0	Sconica_v3_42040-RA	0	0	0	5	0	0
CCAse	AT1G22660	0	0	0	0	0	0	FUN_019134-T1	3	2	0	1	1	0
MTF	AT1G66520	1	0	0	0	0	0	Sconica_v3_07812-RA	7	4	7	10	6	2
TilS	AT3G24560	0	0	0	0	0	0	Chr10-anno1.g29341.t1	1	0	0	0	0	0
tRNase Z1[a]	AT1G74700	0	0	0	0	0	0	FUN_038515-T1	2	0	0	0	0	0
tRNase Z2	AT2G04530	0	0	0	0	0	0	Chr03-anno1.g57321.t1	0	0	1	3	0	0
tRNase Z3	AT1G52160	0	0	0	0	0	0	Multiple	0	0	0	0	0	0
tRNase Z4	AT3G16260	0	0	0	0	0	0	Multiple	0	0	0	0	0	0
PRORP1	AT2G32230	0	0	1	0	0	0	FUN_011644-T1	0	0	2	1	0	0
PRORP2	AT2G16650	0	0	0	0	0	0	No gene found	NA	NA	NA	NA	NA	NA
PRORP3	AT4G21900	0	0	0	0	0	0	No gene found	NA	NA	NA	NA	NA	NA

[a] A second *S. conica* gene had a top BLAST hit to *A. thaliana* tRNase Z1, but no PSMs were detected for it.

Table S4. Genes encoding subunits of the *A. thaliana* mitoribosome (Waltz et al. 2020) and their counterparts in *S. conica*

			Silene PSM Counts						Mito-
**Protein**	***Silene* gene ID**[a]	***Arabidopsis* gene ID**[a]	**Mt1**	**Mt2**	**Cp1**	**Cp2**	**Leaf1**	**Leaf2**	**Enriched**[b]
uL1m	Sconica_v3_19193-RA	AT2G42710	15	11	1	0	0	0	*
uL2m N-term	Chr03-anno2.g23183.t1	***ATMG00560***	6	7	0	0	0	0	
uL2m C-term	Sconica_v3_41290-RA	AT2G44065	9	11	0	0	0	0	*
uL3m	Sconica_v3_37834-RA	AT3G17465	8	8	0	0	0	0	*
uL4m	Sconica_v3_19156-RA	AT2G20060	8	5	0	0	0	1	*
uL5m	***mito rpl5***	***ATMG00210***	6	2	0	0	0	0	*
uL6m	Chr08-anno2.g6040.t1	AT2G18400	0	3	0	0	0	0	
bL9m	FUN_038434-T1	AT5G53070	5	5	0	0	0	0	*
uL10m	Sconica_v3_43288-RA	AT3G12370	6	3	0	0	0	0	*
uL11m	Chr03-anno1.g57427.t1	AT4G35490	7	3	0	0	0	0	*
bL12m	Sconica_v3_40269-RA	AT3G06040	11	12	0	0	0	0	*
uL13m	Chr04-anno1.g50273.t1	AT3G01790	8	5	0	0	0	0	*
uL14m	FUN_046256-T1	AT5G46160	5	4	0	0	0	0	*
uL15m	Sconica_v3_17785-RA	AT5G64670	9	7	0	0	0	0	*
uL16m	Sconica_v3_30744-RA	***ATMG00080***	8	3	0	0	0	0	
bL17m	Sconica_v3_10014-RA	AT5G09770	3	3	0	0	0	0	*
uL18m	Sconica_v3_57073-RA	AT5G27820	7	5	0	0	0	0	*
bL19m	FUN_019669-T1	AT1G24240	6	7	0	0	0	0	*
bL20m	Sconica_v3_23041-RA	AT1G16740	6	5	0	0	0	0	*
bL21m	Sconica_v3_47180-RA	AT4G30930	8	6	0	0	0	0	*
uL22m	Sconica_v3_06702-RA	AT1G52370	9	7	0	0	0	0	*
uL23m	Chr08-anno1.g34018.t1	AT4G39880	8	6	0	0	0	0	*
uL24m	Sconica_v3_17854-RA	AT5G23535	3	5	0	0	0	0	*
bL25m	Sconica_v3_43645-RA	AT5G66860	7	8	0	0	0	0	*
bL27m	Sconica_v3_22742-RA	AT2G16930	3	2	0	0	0	0	
bL28m	Chr10-anno1.g32626.t1	AT4G31460	11	8	0	0	0	0	*
uL29m	Sconica_v3_24808-RA	AT1G07830	4	1	0	0	0	0	
uL30m	Sconica_v3_42097-RA	AT5G55140	3	1	0	0	0	0	*
bL31m	Chr03-anno2.g23587.t1	AT5G55125	2	1	0	0	0	0	*
bL32m	Not annotated	AT1G26740	N/A	N/A	N/A	N/A	N/A	N/A	
bL33m	Sconica_v3_46773-RA	AT5G18790	2	0	0	0	1	0	
bL35m	Chr02-anno1.g23806.t1	AT5G45590	3	2	0	0	0	0	*
bL36m	Chr09-anno2.g40880.t1	AT5G20180	1	0	0	0	0	0	*
mL40	Chr04-anno1.g46970.t1	AT4G05400	3	3	0	1	0	0	*
mL41	FUN_023568-T1	AT5G40080	1	3	0	0	0	0	*
mL43	Chr10-anno1.g28670.t1	AT3G59650	4	2	0	0	0	0	*
mL46	Sconica_v3_41121-RA	AT1G14620	13	9	0	0	1	0	*
mL53	Sconica_v3_53235-RA	AT5G39600	8	3	0	0	0	0	
mL59/mL64	Chr06-anno1.g19483.t1	AT4G22000	6	1	0	0	0	0	
mL60	Sconica_v3_30462-RA	AT1G27435	1	3	0	0	0	0	
mL80	Chr01-anno1.g62765.t1	AT1G73940	6	1	0	0	0	0	*
mL87	Sconica_v3_10159-RA	AT3G51010	3	2	0	0	0	0	*
mL101 (rPPR4)	Sconica_v3_25967-RA	AT1G60770	9	7	0	0	0	0	*
mL102 (rPPR5)	Sconica_v3_09229-RA	AT2G37230	27	23	0	0	0	0	
mL104 (rPPR9)	Sconica_v3_02474-RA	AT5G60960	19	17	0	0	0	0	*
uS1	Sconica_v3_17748-RA	Lost in *Arabidopsis*	9	6	0	0	0	0	*

uS2m	Sconica_v3_17930-RA	AT3G03600	13	7	0	0	0	0	*
uS3m	***mito rps3***	***ATMG00090***	24	17	0	0	0	0	*
uS4m N-term	FUN_045851-T1/FUN_045790-T1	***ATMG00290***	5	7	0	0	0	0	
uS4m C-term	Sconica_v3_40630-RA	***ATMG00290***	10	3	0	0	0	0	*
uS5m	Sconica_v3_25633-RA	AT1G64880	18	20	0	0	0	0	*
bS6m	Sconica_v3_05402-RA	AT3G18760	3	2	0	0	0	0	*
uS7m	Lost in *Silene*	***ATMG01270***	N/A	N/A	N/A	N/A	N/A	N/A	
uS8m	Sconica_v3_37372-RA	AT4G29430	3	4	0	0	0	0	
uS9m	Sconica_v3_19137-RA	AT3G49080	10	5	0	0	0	0	*
uS10m	Sconica_v3_23770-RA	AT3G22300	7	5	0	0	0	0	
uS11m	Sconica_v3_11125-RA	AT1G31817	8	2	0	0	0	0	
uS12m	Sconica_v3_11465-RA	***ATMG00980***	5	2	0	0	0	0	*
uS13m	***mito rps13***	AT1G77750	3	5	0	0	0	0	*
uS14m	FUN_029269-T1	AT2G34520	2	5	0	0	0	0	*
uS15m	Sconica_v3_25605-RA	AT1G15810	19	14	0	0	0	0	*
bS16m	Sconica_v3_11133-RA	AT5G56940	10	5	0	0	0	0	*
uS17m	Sconica_v3_53755-RA	AT1G49400	11	8	0	0	0	0	*
bS18m	Chr02-anno1.g21175.t1	AT1G07210	10	9	0	0	0	0	*
uS19m	FUN_042562-T1	AT5G47320	9	6	0	0	0	0	
bS21m	FUN_011913-T1	AT3G26360	0	0	0	0	0	0	
mS23	Sconica_v3_11989-RA	AT1G26750	16	12	0	0	0	0	*
mS26	Sconica_v3_22695-RA	AT5G49210	4	6	0	0	0	0	*
mS29	Chr04-long_reads1.PB.1859.1	AT1G16870	19	14	0	0	0	0	*
mS33	Sconica_v3_48073-RA	AT5G44710	4	3	0	0	0	0	*
mS34	Chr02-anno1.g21262.t1	AT5G52370	7	8	0	0	0	0	*
mS35	Sconica_v3_02574-RA	AT3G18240	12	7	0	0	0	0	*
mS37	FUN_006674-T1	AT1G47278	5	4	0	0	0	0	*
mS38	Sconica_v3_12210-RA	AT5G63150	0	0	0	0	0	0	
mS41	Sconica_v3_30872-RA	AT5G26800	2	1	0	0	0	0	*
mS45	Chr09-anno1.g45083.t1	AT5G62270	7	8	0	0	0	0	
mS47	Chr08-anno1.g33382.t1	AT4G31810	15	7	0	0	0	0	*
mS80 (rPPR6)	Chr10-anno1.g29827.t1	AT3G02650	21	13	0	0	0	0	*
mS83 (rPPR10)	Sconica_v3_49004-RA	AT4G15640	6	6	0	0	0	0	*
bTHXm	Chr01-anno1.g64414.t1	AT2G21290	0	0	0	0	0	0	

[a] Names in bold italics correspond to genes in the mitogenome. All others are in the nuclear genome.

[b] Asterisks in the Mito-Enriched column indicate significantly greater ($p < 0.05$) normalized MS1 ion intensities in the mitochondrial fraction than in total leaf samples based on a Student's *t*-test (with log-transformed values). Values used in these tests exclude contributions from shared peptides but do include intensities reported only as "Peak Found" without PSM support. The identified differences would be even larger if these were excluded. Missing ion intensity values (i.e., proteins that were not detected at all in a sample) were imputed as values of 1e6, approximating the low end of detection reported for these samples.
